# Don’t panic: IVF efficiency in the USA remains strong

**DOI:** 10.1093/hropen/hoag060

**Published:** 2026-07-02

**Authors:** Micah J Hill, Jennifer F Kawwass, Jennifer L Eaton

**Affiliations:** Shady Grove Fertility Center, Rockville, MD, USA; Department of OBGYN, Emory Reproductive Center, Emory University School of Medicine, Atlanta, GA, USA; Department of OBGYN, Women & Infants Hospital and the Warren Alpert Medical School of Brown University, Providence, RI, USA

Dear Editor,

As leaders of the Society for Assisted Reproductive Technology (SART), we read with great interest the paper from Gleicher and colleagues (Gleicher *et al.*, 2026) that was recently published in *Human Reproduction Open*. The authors use Centers for Disease Control and Prevention (CDC) data from 2012 to 2021 to conclude that IVF efficiency in the USA is declining. The logic for their argument is listed below:

1. The total number of IVF stimulation cycle starts increased by 234%.
2. The total number of embryo transfer cycles did not increase as much: 150%.
3. The difference is partially explained by embryo banking increasing by 902%.
4. Embryo banking is an ‘add-on’ that is overutilized.
5. Therefore, Gleicher *et al.* (2026) conclude that IVF efficiency is dropping in the USA owing to the ‘add-on’ of embryo banking.

The last two points are based on a faulty assumption—that embryo banking is bad for patients. An accurate title for the article would have reflected the increase in embryo banking in the USA. The authors could then have followed with their personal viewpoint that embryo banking is bad for patients. Instead, Gleicher and colleagues create an arbitrary metric to state that IVF efficiency is declining in the USA, a perspective that is only valid if one believes that embryo banking harms patients. We strongly disagree from both ethical and clinical viewpoints. In our opinion, embryo banking is not an IVF ‘add-on’. Rather, embryo banking expands treatment options and future fertility potential.

Importantly, neither SART, the American Society for Reproductive Medicine (ASRM), nor ESHRE defines embryo banking as an ‘add-on’. It is the opposite. The most recent ESHRE Guideline on female fertility preservation states, ‘embryo cryopreservation is an established option for fertility preservation’ (ESHRE Guideline Group on Female Fertility Preservation *et al*., 2020) and that banking is appropriate for ‘fertility preservation […] for women worried about age-related fertility loss’ (ESHRE, 2021). Embryo banking cycles include patients who are banking embryos for medical indications and those aiming to increase their total family size potential. We therefore reject the argument that embryo banking is an add-on.

With this assertion, the question then follows, ‘is IVF cycle efficiency in the USA actually declining?’ When embryo banking cycles are excluded, Table 1 of Gleicher *et al*. (2026) demonstrates that ART cycle efficiency in the USA has been stable over the decade at around 32%. Current CDC and historic SART reporting include all cycles (banking and non-banking) in the denominator for determination of pregnancy. As more patients have legitimate indications for embryo banking, the apparent success of IVF per cycle start declines. Conversely, if these banking cycles are excluded, it becomes clear that the birth per cycle is similar to higher than it has been in the past. It is simultaneously important to note that a new SART Clinic Summary Report (CSR) helps patients understand outcomes per cycle start, including banking (Society for Assisted Reproductive Technology, 2024).

More simply, there are two reasons why CDC and historic SART reporting significantly underestimate ART cycle efficiency in the USA:

1. In the CDC tables and the historic SART tables, if multiple IVF cycles are initiated with the intent to achieve pregnancy while also banking embryos to have more than one child in the future, once an embryo is transferred, the other IVF cycles will count as negative cycles. The registries have historically given a 1-year window for embryo transfer to occur; otherwise, the cycle is counted as negative.
2. In the CDC tables and the historic SART tables, embryo banking is grossly underreported compared to what actually happened.

For the first point, as a practical example, a 38-year-old patient wants three children and performs three IVF cycles to get six embryos. She then transfers the top embryo, which happens to be from Cycle 3, and has a live birth. Using the CDC tables, Cycles 1 and 2 automatically count as a negative, even though those cycles have embryos in storage and have not yet had the chance of transfer. This underestimates IVF cycle efficiency.

For the second point, from 2014 to 2017 SART data, embryo banking was reported in roughly 10% of IVF cycles where embryo banking actually occurred (McCulloh *et al*., 2024). Here, embryo banking was more accurately defined not by the precycle intention, but by what actually happened: an IVF cycle that generated useable embryo(s) in cryostorage without any attempt to perform an embryo transfer within 1 year. These cycles are included in the nationally reported statistics as negative cycles. For this reason, SART updated the annual Clinical Summary Report in 2023 to include reports of what happened at a per cycle level, while also reporting cancellation rates and cycles in which nothing was available to freeze (Society for Assisted Reproductive Technology, 2024).

In summary, we disagree with Gleicher *et al*. (2026) on two fundamental points. First, embryo banking is not an IVF add-on. Embryo banking is a powerful family building option for appropriately counseled patients. Secondly, SART data demonstrate IVF efficiency in the USA is improving and not declining.

Keep calm. Don’t panic.

## Disclosures

The authors have no financial conflicts of interest.
